# Long intergenic non-coding RNA LINC00485 exerts tumor-suppressive activity by regulating miR-581/EDEM1 axis in colorectal cancer

**DOI:** 10.18632/aging.202354

**Published:** 2021-01-10

**Authors:** Chenmeng Li, Bei Pan, Xiangxiang Liu, Jian Qin, Xuhong Wang, Bangshun He, Yuqin Pan, Huiling Sun, Tao Xu, Xueni Xu, Kaixuan Zeng, Shukui Wang

**Affiliations:** 1 School of Medicine, Southeast University, Nanjing 210009, Jiangsu, China; 2 General Clinical Research Center, Nanjing First Hospital, Nanjing Medical University, Nanjing 210006, Jiangsu, China; 3 Jiangsu Collaborative Innovation Center on Cancer Personalized Medicine, Nanjing Medical University, Nanjing 211100, Jiangsu, China

**Keywords:** linc00485, microRNA-581, EDEM1, colorectal cancer

## Abstract

Long non-coding RNAs (lncRNA) play a vital role in colorectal cancer (CRC) progression. To investigate the role of long intergenic non-coding RNA *LINC00485* in CRC, we performed *in vitro* functional experiments. LoVo tumor-bearing and liver metastasis mice were used as *in vivo* models. We found that *LINC00485* expression was significantly lower in CRC tissues and cancer cells than in paired normal samples and human normal colonic epithelial cells. Lower expression of *LINC00485* predicted poor prognosis in CRC patients. *LINC00485* knockdown promoted the proliferation, migration, and invasion of FHC cells, while *LINC00485* overexpression weakened these abilities of LoVo cells. MicroRNA *miR-581* was the downstream target of *LINC00485*, which was downregulated in CRC samples and cancer cells compared to normal tissues and normal colonic epithelial cells. *MiR-581* overexpression induced proliferation, migration, and invasion of FHC cells, while *miR-581* antagomir treatment produced opposite results. *MiR-581* directly targeted the 3'UTR of *EDEM1* and inhibited its expression and induction of epithelial-mesenchymal transition of CRC. In mouse models, *LINC00485* knockdown or down-regulation of *miR-581* significantly repressed CRC cell growth and prevented CRC liver metastasis. Overall, *LINC00485* suppressed CRC tumorigenesis and progression by targeting the *miR-581*/*EDEM1* axis. *LINC00485* may be a potential therapeutic target for CRC.

## INTRODUCTION

Colorectal cancer (CRC) is a common digestive system neoplasm (DSN) that threatens human health globally [1]. Epidemiological statistics have indicated that the morbidity and mortality of CRC rank third and fourth among malignancies, respectively [2]. The pathogenesis of CRC is complex and has not been fully elucidated. Recently, an increasing number of studies have reported that non-coding RNAs, including long non-coding RNAs (LncRNA) [3], microRNA (miRNA) [4], circular RNAs (circRNAs) [5], and PIWI-interacting RNAs (piRNAs) [6], exert important biological functions in CRC; however, additional research is needed to uncover the specific pathological mechanisms involved in cancer proliferation and metastasis.

LncRNAs are a newly discovered class of non-coding transcripts longer than 200 nucleotides in length [7]. Long intergenic non-coding RNAs (lincRNAs), belong to the class of lncRNAs [8], and act on multiple miRNAs through complementary base pairing, thereby modulating the expression of downstream genes [9]. LincRNAs have been widely identified to regulate tumorigenesis and cancer progression [10–12]. *LINC00485* is a newly discovered lincRNA. A genome-wide association study identified a new locus on 12q23.2 (*LINC00485*), which was associated with uterine leiomyoma [13]. In addition, studies in lung adenocarcinoma (LAC) cells have shown that *LINC00485* directly binds to miRNA-195 to up-regulate checkpoint kinase 1 (*CHEK1*) expression, contributing to LAC cell proliferation and cisplatin resistance [14]. By contrast, we found that the expression of *LINC00485* in CRC tissues was significantly down-regulated when compared to adjacent normal tissues. *MiR-581*, a predicted target for *LINC00485*, was aberrantly upregulated in CRC samples. A significant negative correlation was found between *LINC00485* and *miR-581* expression. Interestingly, down-regulated *miR-581* interfered with hepatocellular carcinoma development [15]. However, it remains unclear whether *LINC00485* directly targets *miR-581* and how it exerts biological activities against CRC progression. Hence, we performed a series of *in vitro* and *in vivo* experiments to uncover the role of *LINC00485* and *miR-581* and to provide new insight into the treatment and diagnosis of CRC.

In this study, we found that *LINC00485* worked as a competing endogenous RNA (ceRNA) against *miR-581* thereby up-regulating *EDEM1* expression in CRC cells, thus promoting proliferation, migration, invasion, and the epithelial-mesenchymal transition (EMT) process of CRC cells. *LINC00485* can be used as a therapeutic target for CRC treatment.

## RESULTS

### LINC00485 is down-regulated in CRC tissues and cells

**Figure 1:**
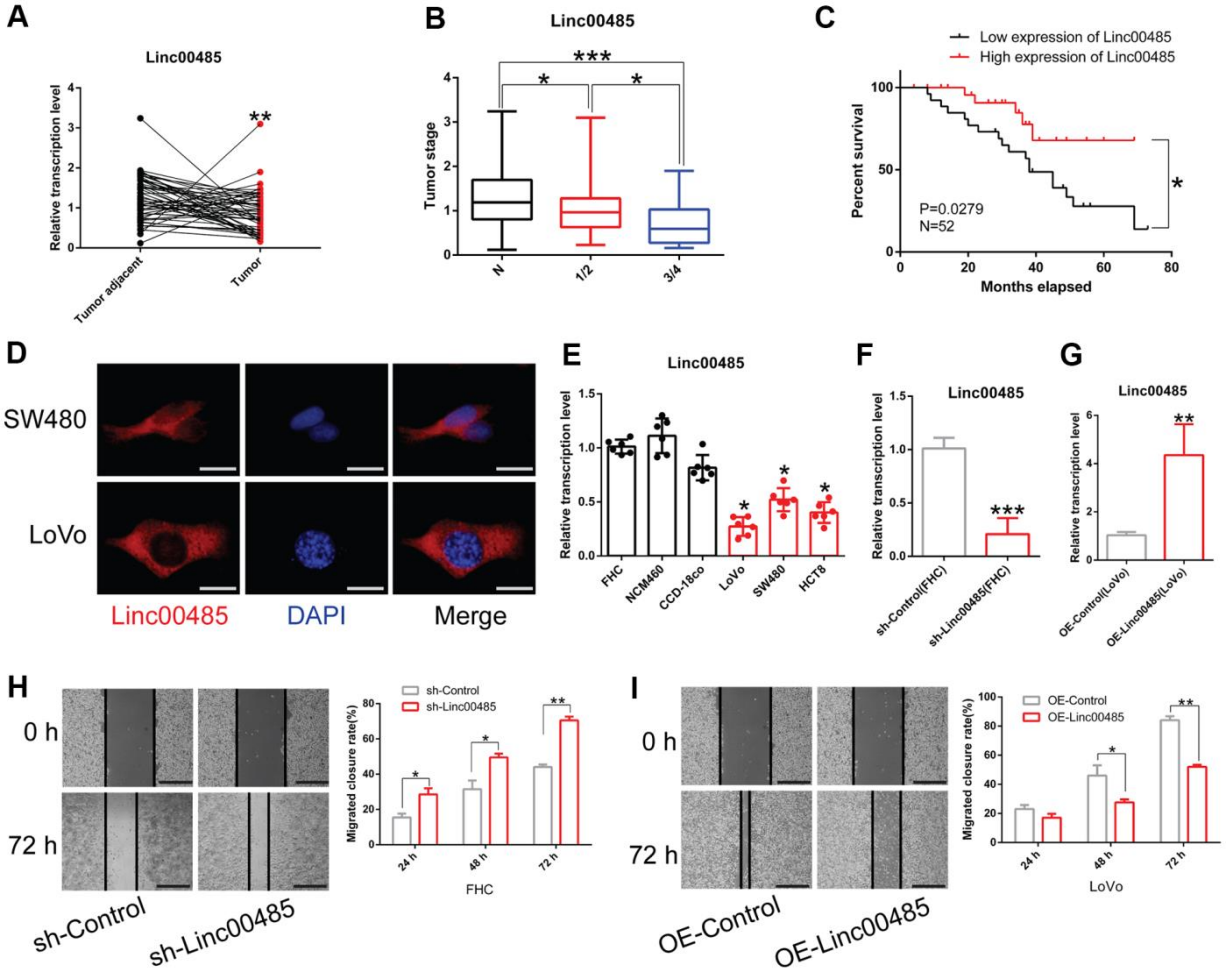
***LINC00485* is downregulated in CRC tissues and cells.** (**A**) *LINC00485* levels in CRC and adjacent normal tissues. (**B**) *LINC00485* levels in CRC patients with stage I/II or III/IV disease is significantly lower than in adjacent normal tissues. (**C**) High expression of *LINC00485* predicts a favorable prognosis of CRC patients. (**D**) The subcellular localization of *LINC00485* in SW480 and LoVo cells was detected by FISH assay. Scale bar, 2 μm. (**E**) *LINC00485* expression is significantly reduced in CRC cells compared to human normal colorectal epithelial cell lines. (**F**) The knockdown efficiency of *LINC00485* RNAi lentivirus in FHC cells. (**G**) *LINC00485* knockdown promotes cell migration in FHC cells. (**H**) *LINC00485* knockdown promotes the migration of FHC cells. (**I**) Overexpression of *LINC00485* suppresses the migratory ability of LoVo cells. Differences between two groups were assessed by applying student’s t-test. Multiple comparison was analyzed using the one-way ANOVA with LSD test. Bars were represented as S.D. *P<0.05; **P<0.01; ***P<0.001. N, normal tissues; sh, short hairpin RNA targeting *LINC00485*.

To understand the role of *LINC00485* in CRC, we collected normal paracancerous tissue and tumor tissues from 52 patients with CRC. We found that the expression of *LINC00485* in CRC tissues was significantly lower than that in adjacent normal tissues (Figure 1A). There was no significant correlation between *LINC00485* expression and clinical parameters such as patient’s age and sex (data not shown), but *LINC00485* expression was strikingly reduced with tumor stage (Figure 1B). Next, lower and higher *LINC00485* expression groups were defined according to median *LINC00485* expression values. The data indicated that CRC patients with higher expression of *LINC00485* survived longer than patients with lower expression of *LINC00485* (Figure 1C). To determine the subcellular localization of *LINC00485* in CRC cells, cells were examined by FISH assay using fluorescent probes. We found that *LINC00485* was mainly expressed in the cytoplasm of CRC cells (SW480, LoVo) (Figure 1D) and human normal colorectal epithelial cells (FHC) (Supplementary Figure 1). Moreover, *LINC00485* levels in CRC cells (LoVo, SW460, HCT8 cell lines) were markedly lower than in human normal colorectal epithelial cell lines (FHC, NCM460, CCD-18co cells) (Figure 1E). We further demonstrated that shRNA-mediated knockdown of *LINC00485* could enhance the migratory ability of FHC cells, while overexpression of *LINC00485* significantly attenuated the migration of LoVo cells (Figure 1F–1I). These findings suggested that *LINC00485* was implicated in CRC progression.

### LINC00485 directly targes microRNA-581

**Figure 2:**
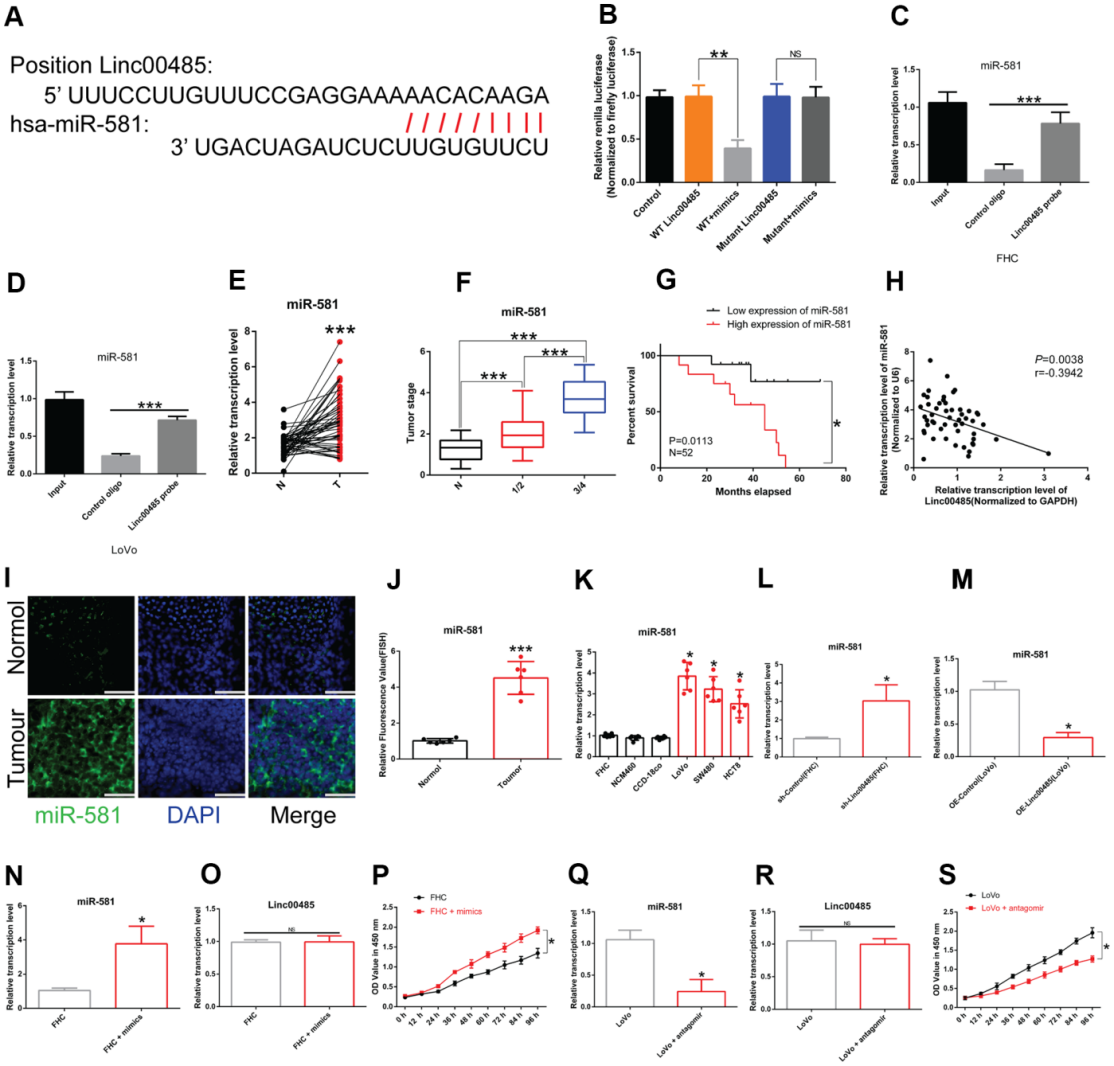
***LINC00485* directly targets *miR-581*.** (**A**) Predicted binding sites between *LINC00485* and *miR-581*. (**B**) The interaction between *LINC00485* and *miR-581* was confirmed by luciferase reporter assays in 293T cells. (**C**, **D**) The RIP assay was performed to validate the interaction between *LINC00485* and *miR-581* in (**C**) FHC cells and (**D**) LoVo cells. (**E**) *miR-581* levels in CRC and adjacent normal tissues. (**F**) *miR-581* levels in CRC patients with stage I/II or III/IV disease is significantly higher than in adjacent normal tissues. (**G**) High expression of *miR-581* predicts poor outcome of CRC patients. (**H**) The expression of *miR-581* is negatively correlated with *LINC00485* level in human tumor tissues. (**I**) The subcellular localization of *miR-581* in CRC and normal tissues. Blue, DAPI; Green, *miR-581*; Scale bar, 50 μm. (**J**) Fluorescence value of *miR-581* expression in tumor and normal tissues. (**K**) *miR-581* expression is significantly elevated in CRC cells compared to the human normal colorectal epithelial cell lines. (**L**) The expression of *miR-581* is significantly elevated in *LINC00485* knockdown FHC cells. (**M**) The expression level of *miR-581* is downregulated in *LINC00485*-overexpressing LoVo cells. (**N**) Transfection efficiency of *miR-581* mimics in FHC cells was determined by RT-qPCR. (**O**) Treatment with *miR-581* mimics has no effect on *LINC00485* expression in FHC cells. (**P**) Treatment with *miR-581* mimics increases FHC cell viability. (**Q**) Transfection efficiency of *miR-581* antagomir in LoVo cells was measured by RT-qPCR. (**R**) Treatment with *miR-581* antagomir has no effect on the expression level of *LINC00485* in LoVo cells. (**S**) *miR-581* knockdown reduces LoVo cell viability. Differences between two groups were assessed by applying student’s t-test. Multiple comparison was analyzed using the one-way ANOVA with LSD test. Bars were represented as S.D. *P<0.05; **P<0.01; ***P<0.001. N, paired normal tissues; T, tumor tissues.

To determine the mechanism underlying the involvement of *LINC00485* in CRC progression, the biological prediction website DIANA-LncBase v2 [16] was used to predict targets of *LINC00485*. The results showed that *miR-581* shared complementary binding sites with *LINC00485* (Figure 2A). Dual-luciferase reporter assays and RNA immunoprecipitation (RIP) analysis confirmed the interaction between *LINC00485* and *miR-581* (Figure 2B–2D). *MiR-581* was highly expressed in CRC tissues compared with normal tissues (Figure 2E) and was significantly associated with tumor stage (Figure 2F). CRC patients with *miR-581* levels above median values have significantly worse prognosis (Figure 2G). We observed a negative correlation between *LINC00485* and *miR-581* in CRC (Figure 2H). Further, our data showed that *miR-581* levels were significantly higher in CRC cell lines and specimens than in human normal colorectal epithelial cell lines and normal samples (Figure 2I–2K). In addition, *LINC00485* knockdown significantly increased the expression of *miR-581* in FHC cells, and the overexpression of *LINC00485* decreased *miR-581* levels in LoVo cells (Figure 2L, 2M). Treatment with *miR-581* mimics or antagomir strongly reduced or elevated *miR-581* levels, neither of which had any effect on *LINC00485* expression; however, *miR-581* knockdown significantly enhanced the cell viability of FHC cells, whereas the overexpression of *miR-581* caused decreased proliferative activity of LoVo cells compared to the matched control cells (Figure 2N–2S).

### LINC00485 regulates cell proliferation, migration, and invasion by acting on miR-581

**Figure 3:**
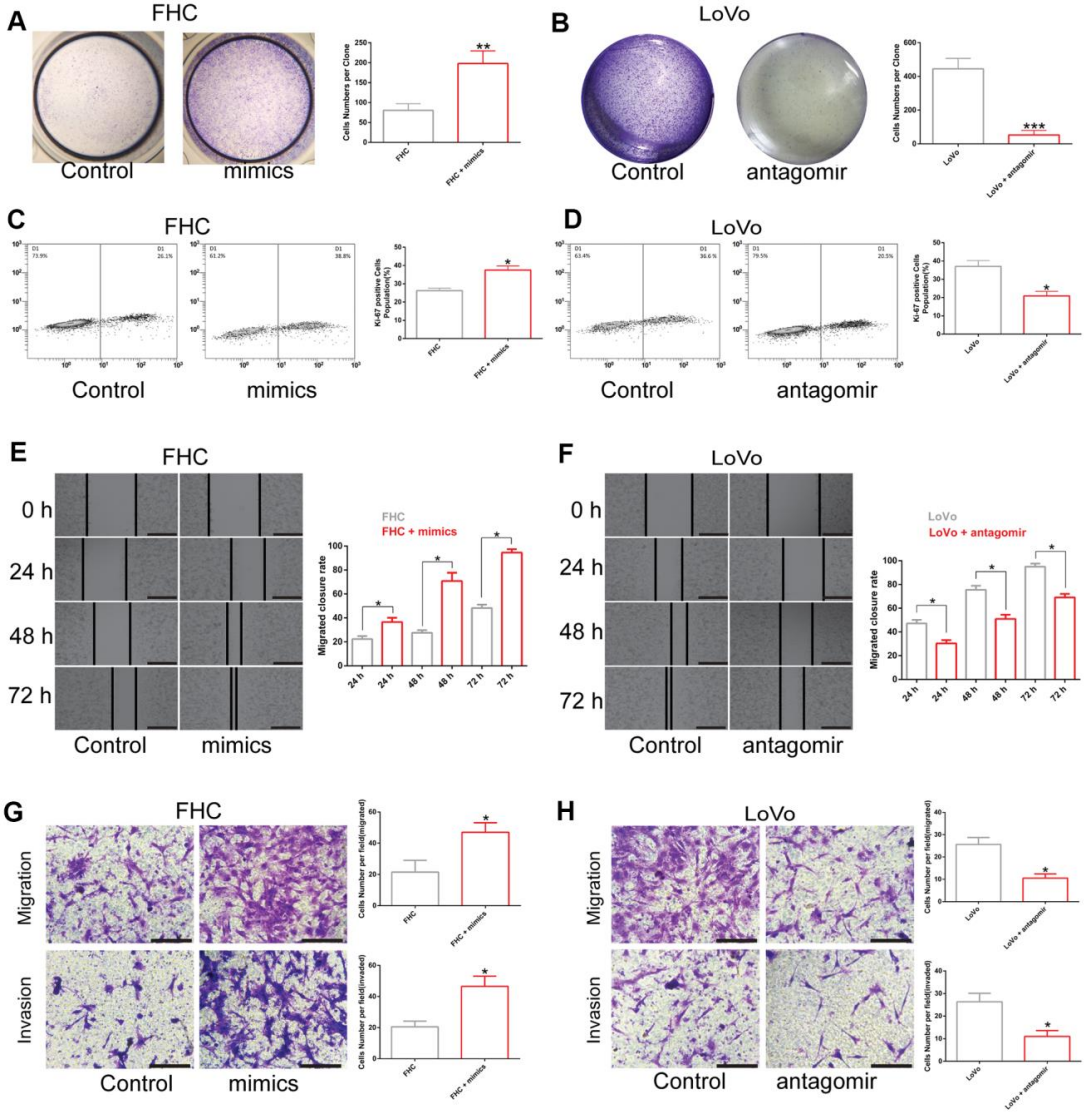
**The effect of *miR-581* on cell proliferation, migration, and invasion.** (**A**, **B**) Colony formation of (**A**) FHC cells transfected with *miR-581* mimics and (**B**) Cell proliferation of LoVo cells transfected with *miR-581* antagomir tested by colony formation assay. (**C**) The percentage of Ki-67-positive FHC cells increases significantly after transfection with *miR-581* mimics. (**D**) The percentage of Ki-67-positive LoVo cells is significantly lower after transfection with *miR-581* antagomir. (**E**, **F**) Wound closure rate of (**E**). FHC cells transfected with *miR-581* mimics and (**F**) LoVo cells transfected with *miR-581* antagomir subjected to the *in vitro* scratch assay. (**G**, **H**) Migratory and invasive abilities of (**G**) FHC cells transfected with *miR-581* mimics and (**H**) LoVo cells transfected with *miR-581* antagomir evaluated by Transwell migration and invasion assays. Comparison between two groups were assessed by student’s t-test. Bars were represented as S.D. *P<0.05; **P<0.01; ***P<0.001.

**Figure 4:**
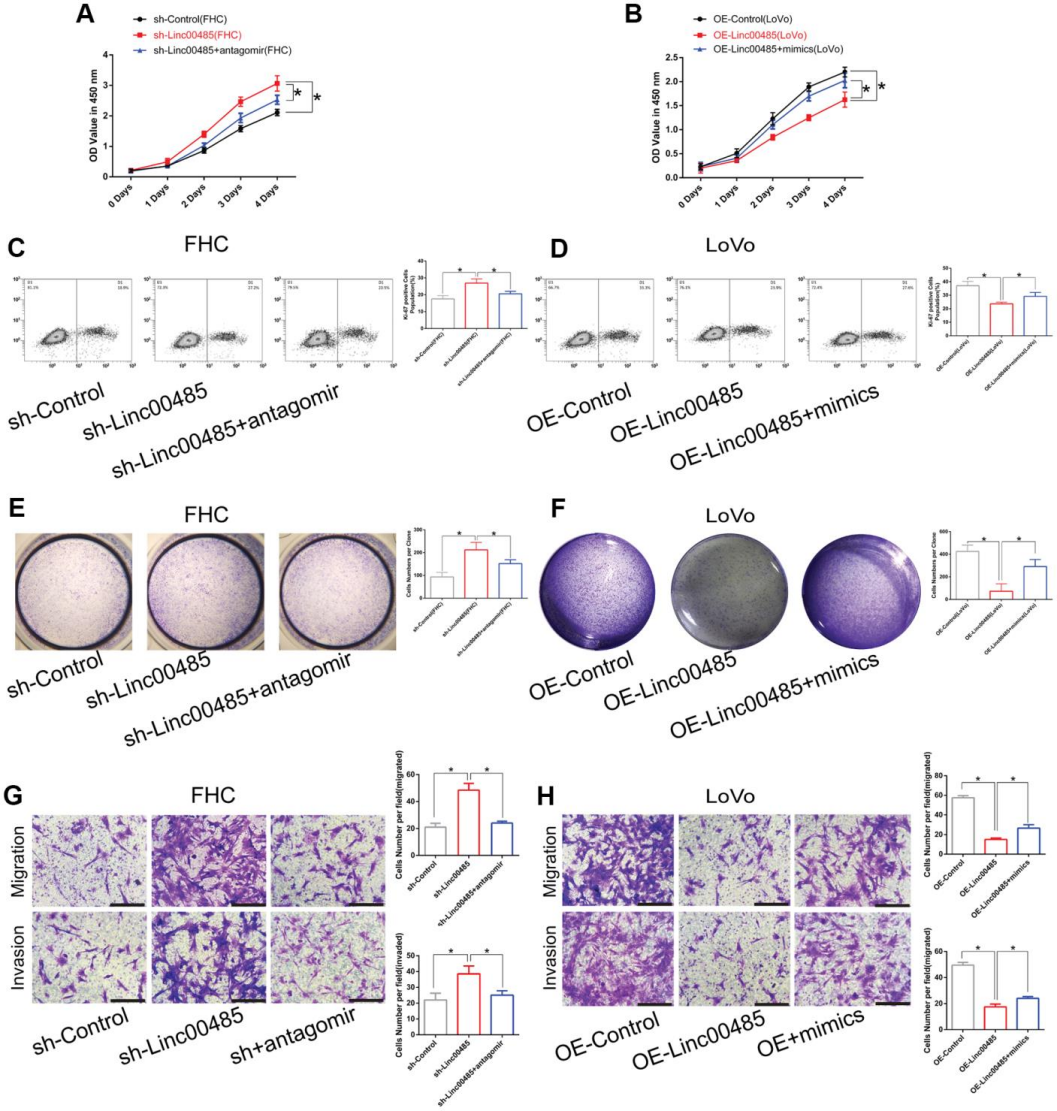
***LINC00485* regulates cell proliferation, migration, and invasion by sponging *miR-581*.** (**A**, **B**) Cell viability of *LINC00485* knockdown FHC cells with or without *miR-581* antagomir treatment for 24 h and (**B**) *LINC00485*-overexpressed LoVo cells with or without *miR-581* mimics treatment for 24 h was evaluated by the CCK-8 assay. (**C**) The percentage of Ki-67-positive cells in (**C**) *LINC00485* knockdown FHC cells with or without *miR-581* antagomir treatment for 24 h was tested by flow cytometry. (**D**) The percentage of Ki-67-positive cells in *LINC00485*-overexpressed LoVo cells with or without *miR-581* mimics treatment for 24 h was evaluated by flow cytometry. (**E**, **F**) Colony forming capability of (**E**) *LINC00485* silenced FHC cells with or without *miR-581* antagomir treatment for 24 h and (**F**) *LINC00485*-overexpressed LoVo cells with or without *miR-581* mimics treatment for 24 h was measured by the colony formation assay. (**G**, **H**) The migratory and invasive abilities of (**G**) *LINC00485*-silenced FHC cells with or without *miR-581* antagomir treatment for 24 h and (**H**) *LINC00485*-overexpressed LoVo cells with or without *miR-581* mimics for 24 h were detected by Transwell assays. Data was analyzed using the one-way ANOVA with LSD test. Bars were represented as S.D. *P<0.05. sh, short hairpin RNA targeting *LINC00485*; OE, overexpression.

To determine how *LINC00485* exerts its functions by regulating *miR-581*, we performed a series of functional experiments *in vitro*. Our findings demonstrated that overexpression of *miR-581* promoted proliferation, migration, and invasion of human normal colorectal epithelial cells (FHC), whereas *miR-581* down-regulation suppressed the proliferative, invasive, and migratory abilities of CRC cells (LoVo) when compared with the matched control group (Figure 3A–3H; Supplementary Figure 2). Furthermore, *LINC00485* silencing significantly elevated the proportion of Ki-67-positive cells, up-regulated the protein expression level of proliferative markers PNCA and Ki-67, arrested cell cycle in Go/G1 phase (Supplementary Figure 3), enhanced colony formation, cell migration, and invasion capabilities of FHC cells in comparison to control cells, which could be partially reversed by *miR-581* silencing (Figure 4A, 4C, 4E, 4G). Additionally, *LINC00485*-overexpressed LoVo cells demonstrated a significant reduction in the percentage of Ki-67-positive cells, the expression levels of PNCA and Ki-67, and in colony formation, proliferative, migratory, and invasive capacities compared with the control groups; however, overexpression of *miR-581* partially abolished *LINC00485* overexpression-induced inhibition of cell proliferation, migration, and invasion in LoVo cells (Figure 4B, 4D, 4F, 4H; Supplementary Figure 3). These findings suggested that *LINC00485* regulated CRC progression by acting via *miR-581*.

### EDEM1 is the downstream molecular target of the LINC00485/miR-581 axis

**Figure 5:**
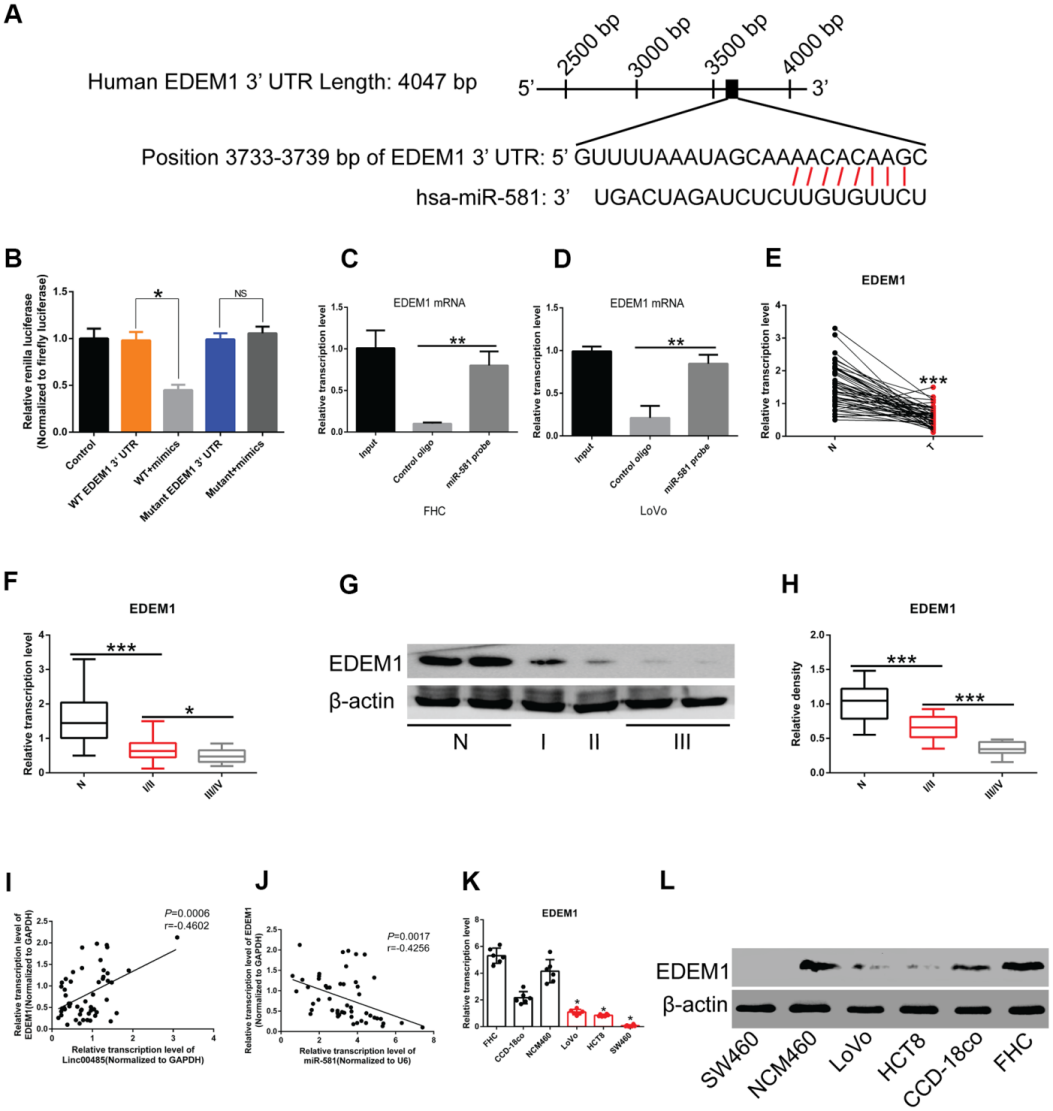
***EDEM1* is the downstream molecular target of the *LINC00485*/*miR-581* axis in CRC cells.** (**A**) Schematic diagram of binding sites of the 3’UTR of *EDEM1* mRNA and *miR-581*. (**B**) Luciferase reporter assay confirming the interaction between the 3’UTR of *EDEM1* mRNA and *miR-581*. (**C**, **D**) RIP assay validating the interaction between the 3’UTR of *EDEM1* mRNA and *miR-581* in (**C**) FHC cells and (**D**) LoVo cells. (**E**) The mRNA level of *EDEM1* is significantly lower in 52 CRC tissues than in paired normal tissues. (**F**, **G**, **H**) The (**F**) mRNA and (**G**, **H**) protein levels of *EDEM1* in CRC patients with stage I/II or III/IV are significantly lower than in adjacent normal tissues. *EDEM1* expression level decreases significantly with the advancing tumor stage. (**I**) A positive correlation between *LINC00485* level and the expression of *EDEM1* in 52 CRC tissues. (**J**) *EDEM1* expression is negatively correlated with *miR-581* levels in 52 CRC tissues. (**K**, **L**) The (**K**) mRNA and (**L**) protein levels of are significantly reduced in CRC cells compared with in human normal colorectal epithelial cell lines. Comparison between two groups were assessed using student’s t-test. Multiple comparison was analyzed using the one-way ANOVA with LSD test. Bars were represented as S.D. *P<0.05; **P<0.01; ***P<0.001. WT, wild type; N, adjacent normal tissues; T, tumor tissues; NS, not significant.

To explore downstream targets of *miR-581*, we performed bioinformatics analysis using TargetScan (http://www.targetscan.org/) to predict messenger RNA (mRNA) targets. We identified the complementary sites between *miR-581* and *EDEM1* (Figure 5A). Dual-luciferase reporter assays revealed that wild-type (WT) *LINC00485*, but not the mutant *LINC00485*, could directly bind to *miR-581*. When *miR-581* was co-transfected with the WT 3'- untranslated region (3'UTR) of *EDEM1* into 293T cells, the luminescence activity was significantly reduced, while when *miR-581* was co-transfected with mutant 3'UTR of *EDEM1* into 293T cells, no differences were observed, indicating that *miR-581* targeted the WT 3'UTR of *EDEM1*, but not the mutant 3'UTR of *EDEM1* (Figure 5B). RIP and real time quantitative polymerase chain reaction (RT-qPCR) experiments further confirmed the binding relationship between *miR-581* and 3'UTR of *EDEM1* mRNA (Figure 5C, 5D). Moreover, by searching The Cancer Genome Atlas (TCGA) dataset, we found that expression of *EDEM1* was significantly down-regulated in CRC tissues compared to adjacent normal tissues (Supplementary Figure 4A). *EDEM1* expression also showed significant differences in correlation with the patient's sex, tumor type, and stage (Supplementary Figure 4B–4D). A further search of the Human Protein Atlas (HPA) database revealed that the overall survival of CRC patients with low *EDEM1* expression was significantly lower than those with high *EDEM1* expression (Supplementary Figure 4E). In our study, levels of *EDEM1* were markedly decreased in CRC tissues compared with normal tissues (Figure 5E), an association that correlated with tumor stage (Figure 5E–5H). *EDEM1* expression was positively correlated with *LINC00485* levels (Figure 5I), while it inversely correlated with *miR-581* expression (Figure 5J). *EDEM1* expression *in vitro* was significantly reduced in CRC cell lines compared to human normal colorectal epithelial cells at both the transcriptional and translational levels (Figure 5K, 5L).

### LINC00485/miR-581/EDEM1 axis regulates epithelial-to-mesenchymal transition in CRC

**Figure 6:**
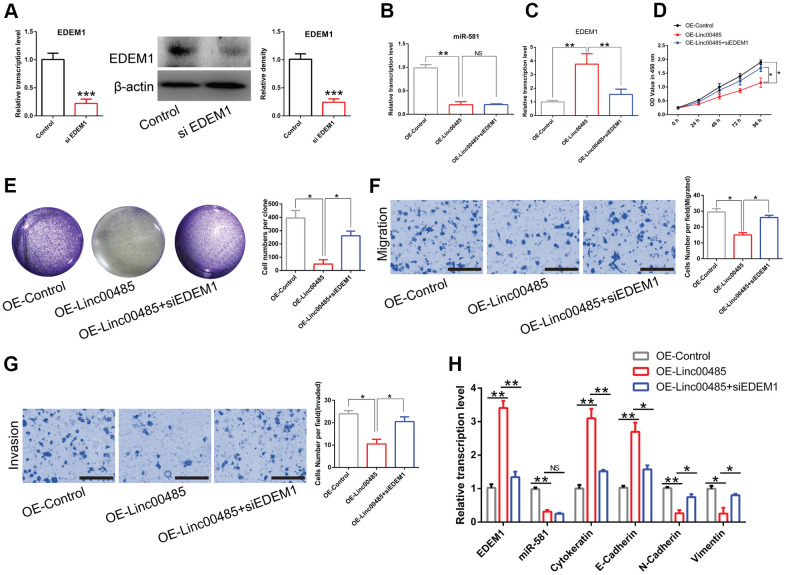
***LINC00485* modulates CRC cell proliferation, migration, and invasion by regulating *miR-581*/*EDEM1* axis.** (**A**) The transfection efficiency of small interfering RNA targeting *EDEM1* was validated by RT-qPCR analysis and western blotting assay. (**B**) *miR-581* levels are significantly down-regulated both in *LINC00485*-overexpressed LoVo cells and *LINC00485*-overexpressed LoVo cells transfected with si*EDEM1*. (**C**) *EDEM1* expression increases in *LINC00485*-overexpressed LoVo cells, but decreases in *LINC00485*-overexpressed LoVo cells after transfection with si*EDEM1*. (**D**) Cell viability of *LINC00485*-overexpressed LoVo cells with or without si*EDEM1* treatment was measured by the CCK-8 assy. (**E**) The colony formation assay results showing that overexpression of *LINC00485* significantly inhibits the colony forming ability of LoVo cell, but *EDEM1* knockdown reverses the result induced by *LINC00485* overexpression. (**F**) Transwell migration and (**G**) invasion assays showing the effect of *EDEM1* knockdown on *LINC00485*-overexpressed LoVo cells. (**H**) The mRNA levels of *EDEM1*, *miR-581*, cytokeratin, E-cadherin, N-cadherin, and vimentin in *LINC00485*-overexpressed LoVo cells transfected with or without si*EDEM1*. Comparison between two groups were assessed using student’s t-test. Multiple comparison was analyzed using the one-way ANOVA with LSD test. Bars were represented as S.D. *P<0.05; **P<0.01; ***P<0.001. NS, not significant; OE, overexpression; si, small interfering RNA targeting EDEM1; EDEM1, ER-degradation-enhancing alpha-mannosidase-like protein-1.

To unravel whether the *LINC00485*/*miR-581* axis exerts its biological function by modulating *EDEM1* expression, small interfering RNA (siRNA) targeting *EDEM1* were used to inhibit *EDEM1* expression in LoVo cells, which resulted in a significant reduction of *EDEM1* expression compared to the untransfected cells (Figure 6A). In *LINC00485*-overexpressing LoVo cells, *miR-581* levels were significantly down-regulated; however, further treatment with si*EDEM1* had no effect on *miR-581* expression (Figure 6B), but markedly reduced *EDEM1* expression as expected (Figure 6C). Consistent with previous results, *LINC00485* knockdown exhibited significant suppressive activity in LoVo cells. Transfection si*EDEM1* partially counteracted *LINC00485* knockdown-induced reduction of cell proliferation (Figure 6D), colony formation (Figure 6E), migration (Figure 6F), and invasion (Figure 6G) abilities of LoVo cells compared with the untransfected LINC0048-knockdown cells. These results confirmed that *LINC00485* exerted its effects by regulating the *EDEM1*/*miR-581* axis. EMT contributes to the pathogenesis of cancer metastasis [17]. In this cellular context, the expression of epithelium-related genes (cytokeratin, E-cadherin) was significantly upregulated, while that of mesenchymal-associated genes (N-cadherin, vimentin) was downregulated in *LINC00485*-overexpressed LoVo cells compared to control cells. Further knockdown of *EDEM1* using siRNA, could partially reverse the changes caused by *LINC00485* overexpression (Figure 6H).

**Figure 7:**
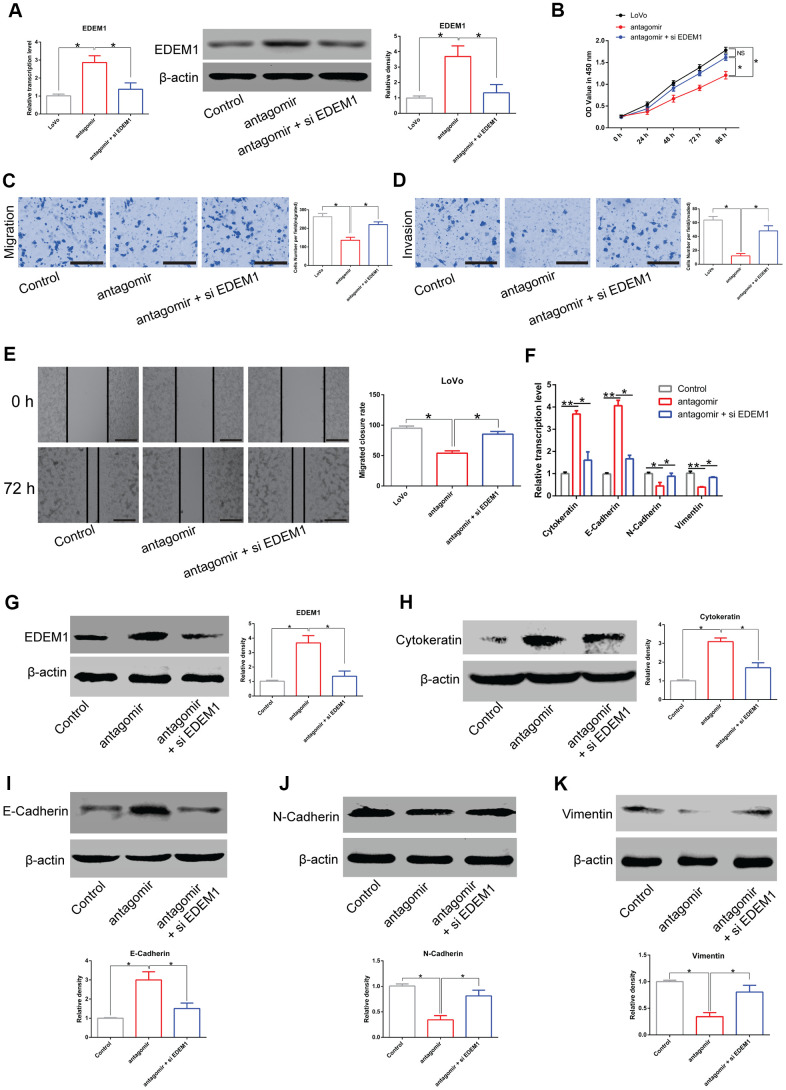
***LINC00485*/*miR-581*/*EDEM1* axis regulates epithelial to mesenchymal transition in CRC cells.** (**A**) The expression of *EDEM1* in LoVo cells transfected with *miR-581* antagomir or in combination with si*EDEM1*. (**B**) Cell viability of LoVo cells transfected with *miR-581* antagomir or in combination with si*EDEM1* measured by the CCK-8 assay. (**C**–**E**) The (**C**, **E**) migratory and (**D**) invasive capabilities of LoVo cells transfected with *miR-581* antagomir or co-transfected with *miR-581* antagomir and si*EDEM1* were assessed by Transwell migration and invasion assays and the *in vitro* scratch assay. (**F**) The mRNA levels of cytokeratin, E-cadherin, N-cadherin, and vimentin in LoVo cells transfected with *miR-581* antagomir or in combination with si*EDEM1* were measured by RT-qPCR. (**G**–**K**) The protein levels of (**C**) *EDEM1*, (**D**) Cytokeratin, (**E**) E-cadherin, (**F**) N-cadherin and (**G**) Vimentin in LoVo cells transfected with *miR-581* antagomir or in combination with si*EDEM1* were quantified by western blotting assay. Data were analyzed using one-way ANOVA with LSD test. Bars were represented as S.D. *P<0.05; **P<0.01; NS, not significant; OE, overexpression; si, small interfering RNA targeting EDEM1; EDEM1, ER-degradation-enhancing alpha-mannosidase-like protein-1.

In addition, treatment with *miR-581* antagomir increased expression of *EDEM1* in LoVo cells compared to the untreated cells. However, *EDEM1* expression was strikingly attenuated in LoVo cells co-transfected with *miR-581* antagomir and si*EDEM1* compared with LoVo cells transfected with *miR-581* antagomir alone (Figure 7A). In line with the aforementioned results, the knockdown of *miR-581* expression suppressed the proliferation, migration, and invasion of LoVo cells in comparison to the control cells. Finally, treatment with si*EDEM1* was capable of partially rescuing the phenotypes caused by *miR-581* knockdown in LoVo cells (Figure 7B–7E).

Meanwhile, *miR-581* antagomir treatment enhanced the expression of epithelial markers E-cadherin and cytokeratin, but down-regulated the expression of mesenchymal markers N-cadherin and vimentin in LoVo cells at both the transcriptional and translational levels (Figure 7F–7K). Of note, our rescue experiments showed that si*EDEM1* treatment could partially reverse the above-mentioned changes in gene expression induced by the knockdown of *miR-581* (Figure 7F–7K). Cumulatively, these data further supported our findings that the *LINC00485*/*miR-581*/*EDEM1* axis regulated CRC progression.

### Overexpression of LINC00485 or miR-581 knockdown attenuated CRC cell growth and liver metastasis *in vivo*

**Figure 8:**
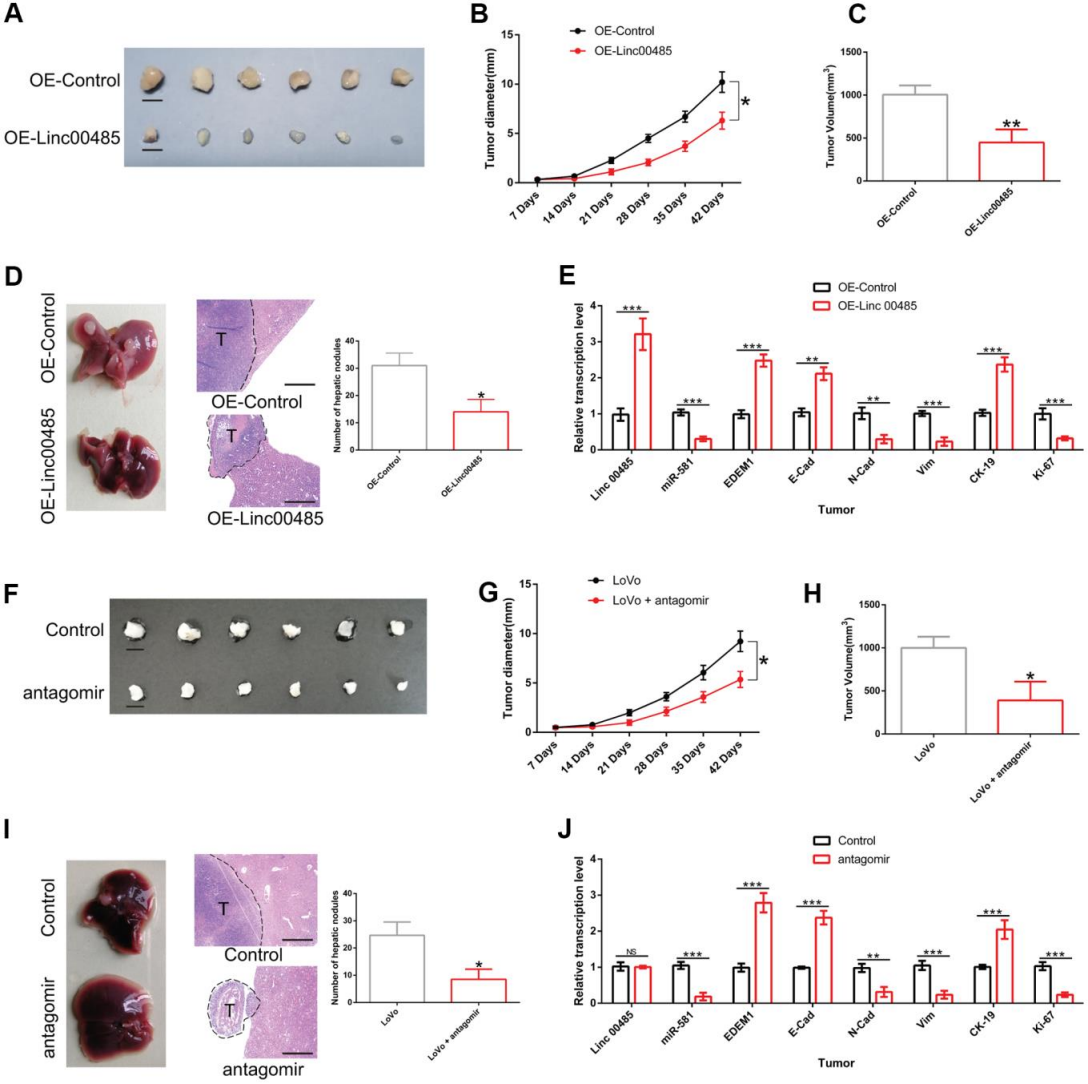
**The effects of *LINC00485* overexpression and *miR-581* knockdown on CRC cell growth and liver metastasis *in vivo*.** (**A**) Representative images of tumors derived from *LINC00485*-overexpressed LoVo cells at day 42 after subcutaneous injection (n=6 per group). (**B**) Tumor diameter was measured weekly after subcutaneous of injection LINC0048-overexpressed LoVo cells into the dorsal side of nude mice (n=6 per group). (**C**) Tumor volume of CRC tissues derived from *LINC00485*-overexpressed LoVo cells detected at day 42. (**D**) The number of hepatic nodules were counted in paraffin sections of liver at day 28 after injection with *LINC00485*-overexpressed LoVo cells into the spleen of the mice (n=6 per group). (**E**) The mRNA expression levels of *LINC00485*, *miR-581*, *EDEM1*, cytokeratin, E-cadherin, N-cadherin, and vimentin in hepatic nodules following injection of *LINC00485*-overexpressed LoVo cells into the spleen of the mice (n=6 per group). (**F**) Representative images of tumors derived from LoVo cells at day 42. Nude mice bearing xenograft tumors received *miR-581* antagomir once a week (n=6 per group). (**G**) Tumor diameter in LoVo-bearing nude mice with administration of *miR-581* antagomir once a week (n=6 per group). (**H**) Tumor volume of LoVo-bearing nude mice with administration of *miR-581* antagomir was evaluated at day 42. (**I**) The number of hepatic nodules in a CRC liver metastases mouse model at day 28 after injection of LoVo cells into the spleen of the mice (n=6 in each group). Mice received *miR-581* antagomir once a week for 4 weeks. (**J**) The mRNA expression levels of LINC00485, *miR-581*, EDEM1, cytokeratin, E-cadherin, N-cadherin, vimentin and Ki-67 in hepatic nodules in a CRC liver metastases mouse model at day 28 after injection of LoVo cells into the spleen of the mice (n=6 in each group). Then, the mice received *miR-581* antagomir once a week for 4 weeks. Data were analyzed using student’s t-test. Bars were represented as S.D. *P<0.05; **P<0.01; ***P<0.001. OE, overexpression.

We established a xenograft nude mouse model and liver metastasis model to investigate the effects of *LINC00485* and *miR-581* on CRC cell growth and metastasis *in vivo*. As described in the Methods section, tumor growth was monitored weekly. When the diameter of the largest tumor reached 1 cm, the nude mice met the humane endpoints of the study. All animals were sacrificed under anesthesia. Tumors were excised and the volumes were measured. We found that overexpression of *LINC00485* significantly decreased tumor diameter, tumor volume at day 42, and the number of hepatic nodules in comparison to the control group, suggesting that the up-regulation of *LINC00485* suppressed tumor growth and liver metastasis of CRC cells (Figure 8A–8D). Additionally, *miR-581* antagomir injection also contributed to the decrease of tumor diameter, tumor volume at day 42, and the number of metastatic nodules of LoVo cells, indicating that *miR-581* knockdown restricted CRC cell growth and liver metastasis *in vivo* (Figure 8F–8I). Furthermore, the expression levels of epithelium markers (cytokeratin, E-cadherin), mesenchymal markers (N-cadherin, vimentin), and cell proliferation markers (CK-19, Ki-67) were evaluated by RT-qPCR in metastatic nodules of nude mice. We found that the results were consistent with *in vitro* observations (Figure 8E, 8J). Overall, it can be concluded that the *LINC00485*/*miR-581*/*EDEM1* axis may represent an important mechanism involved the malignant progression of CRC.

## DISCUSSION

Herein, we reported the role of *LINC00485* in CRC progression. *LINC00485* is downregulated in CRC tissues and cancer cells (LoVo, SW480, HCT8) compared with paired normal samples and human normal colonic epithelial cells (FHC, NCM460, CCD-18co). The *LINC00485*/*miR-581*/*EDEM1* regulatory axis promotes the proliferation, migration, invasion, and EMT of CRC cells. Further, using the xenograft nude mouse model, we found that *LINC00485* knockdown or downregulation of *miR-581* not only significantly repressed CRC cell growth, causing a decrease in tumor volume, but also prevented CRC liver metastasis. Our findings suggested that *LINC00485* plays an important role in CRC progression by regulating the *miR-581*/*EDEM1* axis.

CRC is a serious threat to human health, and leads to hundreds of thousands of deaths annually [1, 18]. Although there are many studies on the pathogenesis of CRC [19–22], the molecular mechanisms underlying CRC are still unknown. Hence, elucidating the biological functions of genes and molecules involved in the occurrence and development of CRC is of benefit for drug development and targeted treatments.

Cancer cell proliferation is one of the ten malignant hallmarks of malignancy [23], and tumor metastasis is the main cause of death in patients with cancer [24]. Mounting evidence has shown that non-coding RNAs, including lincRNA [25] and miRNA [26, 27], exhibit antitumor effects by inhibiting the proliferation of CRC cells. In the present study, we found that low expression of *LINC00485* promoted the proliferation, migration, and invasion of CRC cells, which are distinct features of malignant tumor cells [23]. Moreover, *LINC00485* overexpression significantly restricted tumor growth and liver metastasis of colorectal carcinoma *in vivo*. These results indicated that aberrant expression of *LINC00485* in CRC may play an important role in CRC progression. Conversely, it has been reported that *LINC00485* is overexpressed in LAC cells and that it exerted its functional activity by targeting the *miR-195/CHEK1* axis [14]; therefore, we suspected that *LINC00485* may have a characteristic tissue-specific expression. Further work is required to elucidate the molecular mechanisms underlying this phenomenon.

LincRNAs are mostly found in the cytoplasm [14, 28] and exert their biological activities by sponging miRNA [8]. Herein, our results confirmed that *LINC00485* was predominantly localized within the cytoplasm and acted directly on *miR-581*. Lower expression of *LINC00485* contributed to the upregulation of *miR-581* in CRC, promoting the proliferation, migration, and invasion of CRC cells, which was consistent with previous studies indicating that the up-regulation of *miR-581* regulated the SMAD7/TGFβ signaling pathway, driving CRC metastasis [29]. Further, miRNAs regulate the translation of mRNAs through direct base pairing with specific sites present in the 3’UTR of mRNA [30, 31]. In this study *miR-581* directly targeted the 3’UTR of *EDEM1* mRNA. *EDEM1* is a crucial regulator of the endoplasmic reticulum (ER)-associated degradation (ERAD) pathway, which recognizes N-glycans on misfolded proteins through the mannosidase-like domain (MLD), resulting in the degradation of misfolded glycoproteins [32–34]. The accumulation of misfolded glycoproteins was shown to induce ER stress followed by the activation of autophagy [35]. Autophagy is an important mechanism that the cell utilizes to sustain tumor cell metabolism [36].

The EMT process is closely linked to tumor progression and metastasis [17, 37, 38], and involves epithelial cells losing epithelial characteristics and gaining a mesenchymal phenotype. Based on our results, overexpression of *LINC00485* significantly down-regulated the expression of mesenchymal markers N-cadherin and vimentin, whereas the expression levels of epithelial markers E-cadherin and cytokeratin were upregulated by directly modulating the *miR-581*/*EDEM1* axis, suggesting that cancer cells lose their malignant phenotype. *LINC00485* has multiple roles in the pathogenesis of CRC. Other mechanisms including CRC development and progression merit further investigation.

In conclusion, our findings identified that *LINC00485* was downregulated in CRC tissues in comparison with adjacent normal tissues. Low expression of *LINC00485* was associated with poor prognosis of CRC patients. Downregulation of *LINC00485* contributed to decreased expression of *EDEM1* by sponging of *miR-581*, thereby facilitating CRC cell proliferation, migration, invasion, and the processed of EMT. We show that the *LINC00485*/*miR-581*/*EDEM1* regulatory axis is implicated in regulating the malignant phenotypes of CRC cells both *in vivo* and *in vitro*. Our findings contribute to the elucidation of the molecular pathogenesis underlying CRC.

## MATERIALS AND METHODS

### Patient samples

We collected 52 matched samples (surgically excised tumor tissues and adjacent healthy tissues) from CRC patients with no other medical illnesses, comorbidities, or undergoing neoadjuvant chemoradiotherapy prior to surgery. The detailed information on the enrolled CRC patients is listed in Table 1. Tissues were frozen and rapidly stored in liquid nitrogen after surgical removal. Written informed consent was obtained from patients enrolled in this study. This study was approved by the Ethics Committee of Nanjing First Hospital, Nanjing Medical University.

**Table 1:** Clinical parameters of patients with colorectal cancer (n = 52).

**Clinicopathological characteristics**	**Cases (%)**
Age	
≤60	31 (59.62)
>60	21 (40.38)
Gender	
Male	37 (71.15)
Female	15 (28.85)
TNM stage	
I/II	29 (55.77)
III/IV	23 (44.23)
Lymph node metastasis	
Yes	28 (53.85)
No	24 (46.15)
Tumor invasion depth	
T1/T2	30 (57.69)
T3/T4	22 (42.31)

TNM, tumor node-metastasis.

### Cell culture

Human normal colorectal epithelial cell lines CCD-18co, NCM460, FHC, and human CRC cell lines SW460, HCT8, LoVo were purchased from the Cell Bank of Type Culture Collection of Chinese Academy of Sciences (Shanghai, China). CRC cells and CCD-18co cells were cultured in Dulbecco's modified Eagle's medium (DMEM) (Gibco, Thermo Fisher Scientific, USA) containing 10% fetal bovine serum (FBS) (Gibco) and 1% penicillin-streptomycin (Gibco). NCM460 cells were cultured by McCoy's 5A (modified) Medium (Gibco) with 10% FBS and 1% penicillin-streptomycin. FHC cells were cultured in DMEM/F12 medium (Gibco) supplemented with 10% FBS, 10 ng/mL cholera toxin, 10 mM 4-(2-hydroxyethyl)-1-piperazineethanesulfonic acid (HEPES), 10 mM insulin, 100 ng/mL hydrocortisone, and 10 mM transferrin.

### Establishment of cell lines overexpressing or silencing LINC00485

To construct the *LINC00485*-overexpressing cell line, mRNA of *LINC00485* was reverse transcribed into cDNA and then cloned into the multicloning site of the pLVX plasmid (Sangon Biotech Co., Ltd., Shanghai, China) using cloning primers that incorporated an XhoI site (5’-CCCTCGACTGCGCCCGAGAGGCAGCG-3’) and a PstI site (5’-AACTGCAAACTAGGTCATCTGTTTATT-3’). The construct was subsequently, co-transfected with the packaging plasmids (Sangon Biotech Co., Ltd.) into 293T cells to produce virus particles using the Lipofectamine 3000 transfection reagent (Invitrogen). The virus was collected and mixed in the presence of polybrene (Sangon Biotech Co., Ltd.) to infect LoVo cells. Afterwards, 10 μg/mL of puromycin was used to screen stably expressing cells for 3 days. For the construction of the *LINC00485* knockdown cell line, FHC cells were infected with the *LINC00485* RNAi lentivirus (Genechem Incorporation, Shanghai, China). The shRNA sequence targeting *LINC00485* was as follows: 5’-AATAACCAACCCTATAAACAT-3’.

### Cell transfection

Lipofectamine 2000, miR-581 mimics (5’-UGACUAGAUCUCUUGUGUUCU-3’), miR-581 antagomir (5’-AGAACACAAGAGAUCUAGUCA-3’) and siEDEM1 (5’-UAUUCUGUGAGCAGAAAGGAG-3’) were obtained from Sangon Biotech Co. Ltd. A concentration of 50 nM mimics or antagomir or si*EDEM1* was transiently transfected into FHC or LoVo cells using Lipofectamine 2000 according to the manufacturer’s protocol.

### Construction of reporter vectors and luciferase assay

Dual-luciferase reporter assays were performed using the Dual-Luciferase Assay Kit (TransGen Biotech Co., Ltd., Beijing, China), and Renilla luciferase activity served as an internal control for transfection efficiency. The WT 3'UTR of *EDEM1* or WT *LINC00485* (the binding sites with *miR-581*) was cloned into pGL3 luciferase reporter vector. Primers for LINC00485 were as follows: the forward primer incorporated the PstI restriction site (5’-AACTGCAGAGAGGCAGCGCTCAGACAGC-3’); the reverse primer incorporated the EcoRI restriction site (5’-CGGAATTCTCCGACTTCCAGTTGGGTTC-3’). The primers for cloning the *EDEM1* 3’UTR fragment were established as follows: the forward primer incorporated the XbaI restriction site (5’-CTAGTCTAGATTCCTGTCCATTCCCAGCAT-3’); the reverse primer incorporated the XhoI restriction site (5’- CTAGTCTAGACGTAAGTTTTCCCAGAGTTTCCA-3’). The amplified product (4047 bp) of *EDEM1*was cloned into the pGL3 vector to construct the 3'UTR reporter vector.

Additionally, a high-fidelity amplification kit (Fast Mutagenesis System, #FM111-01, TransGen Biotech Co., Ltd., Beijing, China) and point mutation primers were used to amplify the above plasmid to obtain mutated *EDEM1* 3'UTR vectors. N, N'-dimethyltryptamine (DMT) was applied to screen the mutated *EDEM1* 3’UTR reporter plasmid. Empty plasmid pGL3 was used as the control. Luciferase reporter vectors containing mutated *LINC00485* were also constructed. Afterwards, as the experiment design showed, the corresponding vectors were co-transfected with *miR-581* mimics into 293T cells. Luciferase activity was measured by CLARIOstar^®^ Microplate Reader (BMG Labtech Inc., Germany) at 48 h post transfection.

### Fluorescence *in situ* hybridization assay

The Fluorescence *in situ* hybridization (FISH) assay was used to analyze the subcellular localization of RNA. The corresponding probes were synthesized by Genechem (Shanghai Genechem Co., Ltd., China) based on the sequences of *LINC00485* and *miR-581*. Tissue sections or culturing LoVo cells on sterile glass slides were fixed by immersing slides in 4% formaldehyde for 10 min at room temperature. Subsequently, the slides were treated with pre-chilled PBS containing 0.4% Triton X-100 and 10 μM vanadyl ribonucleoside complexes (VRC) for 30 min at room temperature following hybridization with Cy5-(red, indole dicarboxylic cyanide) labeled *LINC00485* probes or incubation with FAM-labeled miRNA-specific probes (green, carboxy fluorescein) at 37° C for 12 h. After staining the nuclei with 4',6-diamidino-2-phenylindole (DAPI), images were captured by fluorescence microscopy (Olympus, Japan).

### RNA immunoprecipitation experiment

After lysis of the cells according to the instructions of the Magna RIP™ RNA-binding protein Immunoprecipitation Kit (Millipore, Stafford, VA, USA), the cell lysates were incubated with biotin-labeled *LINC00485* for 2 h at 4° C. Biotin-labeled nonsense oligonucleotides were used as negative controls. Streptavidin magnetic beads were incubated for 12 h at 4° C to capture the hybridized nucleic acids. After precipitation by magnetic separation, TRIzol was used to dissolve the precipitate for RNA extraction. Reverse transcription and RT-qPCR assays were then performed to determine the expression levels of *miR-581*.

### Cell counting kit-8 assay

The cells were cultured using a 96-well plate with a transparent bottom (Corning Inc., USA). Cell proliferation was determined following the manufacturer's guidelines. Briefly, after incubation with 10% (CCK-8) reagent (Beyotime Biotechnology, Shanghai, China) at 37° C, absorbance at 450 nm was detected at different time points. The absorbance value was used to evaluate cell proliferative ability.

### Colony formation assay

Cells (1 × 10^3^) were seeded into 6-well dishes, and then cultured for 10 days. After being fixed in 4% paraformaldehyde, the cells were stained with 0.1% Crystal Violet Staining Solution (Beyotime Biotechnology) for 30 min at room temperature followed by observation under the microscope (Leica, Germany). The number of cell colonies were quantified using ImageJ v1.8.0 from ten randomly selected fields.

### Detection of Ki-67 by flow cytometry

Cells were collected and fixed with 4% paraformaldehyde for 30 min following treatment with 0.2% Triton X-100 for 30 min at room temperature. Thereafter, the cells were incubated with anti-Ki-67 antibody (dilution 1:300, #9129S, Cell Signaling Technology, USA) prepared by 5% bovine serum albumin (BSA) solution overnight at 4° C. After coupling with the fluorescent-labeled secondary antibody (dilution 1:500, #4412S, Cell Signaling Technology), fluorescence activity was detected by flow cytometry. Results were analyzed using Flow Jo 7.6.1 (Becton, Dickinson and Company, USA).

### RNA preparation and quantitative PCR (qPCR)

Total RNA was isolated and purified using TRIZol reagent (Thermo Fisher Scientific, Inc., USA) according to the manufacturer's instructions. Mir-X miRNA First-Strand Synthesis Kit (Catalog No. 638315, Takara Biomedical Technology (Beijing) Co., Ltd., China) and Mir-X miRNA quantitative real time polymerase chain reaction (qRT-PCR) TB Green^®^ Kit (Catalog No. 638316, Takara Biomedical Technology (Beijing) Co., Ltd.) were used for miRNA detection. The thermocycling conditions for to detect miRNA expression were as follows: 95° C for 30 s, followed by 40 cycles at 95° C for 15 s, 60° C for 60 s, and 72° C for 10 s. PrimeScript™ RT Master Mix (Perfect Real Time) (Catalog No. RR036A, Takara Biomedical Technology (Beijing) Co., Ltd.) and One Step TB Green^®^ PrimeScript™ RT-PCR Kit (Perfect Real Time) (Catalog No. RR066A, Takara Biomedical Technology (Beijing) Co., Ltd.) were used to evaluate gene expression. RT-qPCR thermocycling parameters to detect gene expression and lncRNA levels were 95° C for 30 s, followed by 40 cycles at 95° C for 15 s, 60° C for 30 s, and 72° C for 10 s. U6 and β-actin served as the internal controls for miRNA levels and gene expression, respectively.

All primers were synthesized by Sangon Biotech Shanghai Company and were showed as follows: β-actin forward: 5’-CCTCGCCTTTGCCGATCC-3’, reverse: 5’-GGATCTTCATGAGGTAGTCAGTC-3’; Cytokeratin forward: 5’-ACCAAGTTTGAGACGGAACAG-3’, reverse: 5’-CCCTCAGCGTACTGATTTCCT-3’; Vimentin forward: 5’-GCCCTAGACGAACTGGGTC-3’, reverse: 5’-GGCTGCAACTGCCTAATGAG-3’; EDEM1 forward: 5’-CGGACGAGTACGAGAAGCG-3’, reverse: 5’-CGTAGCCAAAGACGAACATGC-3’; E-Cadherin forward: 5’-CGAGAGCTACACGTTCACGG-3’, reverse: 5’-GGGTGTCGAGGGAAAAATAGG-3’; N-Cadherin forward: 5’-TCAGGCGTCTGTAGAGGCTT-3’, reverse: 5’-ATGCACATCCTTCGATAAGACTG-3’; Linc00485 forward: 5’-CTGATACATCGCTACTTCTG-3’, reverse: 5’-GTAATCTAACTACTCACACTA-3’; hsa-miR-581 forward: 5’-GAUCUCUUGUGUUCU-3’, reserve: 5’-ATACCTCGGACCCTGCACTG-3’; U6 forward: 5’-CTCGCTTCGGCAGCACA-3’, reverse: 5’-AACGCTTCACGAATTTGCG-3’.

### Western blotting analysis

Total protein was extracted using RIPA lysis buffer (Beyotime Biotechnology). The concentration of proteins was quantified using the bicinchoninic acid (BCA) assay (Beyotime Biotechnology). Proteins were further separated by 10% sodium dodecyl sulfate-polyacrylamide gel electrophoresis (SDS-PAGE) and then transferred onto polyvinylidene difluoride (PVDF) membranes (Millipore, Bedford, MA, USA). Afterwards, the membranes were incubated with the primary antibodies overnight at 4° C before blocking with 5% nonfat dry milk, washed three times with Tris buffered saline with tween (TBST), and then incubated with horseradish peroxidase-conjugated secondary antibodies for 2 h at room temperature. Blots were visualized using BeyoECL Plus (Beyotime Biotechnology). The antibodies used in this study were those listed below: mouse anti-E-Cadherin (dilution 1:1000, #14472, Cell Signaling Technology), mouse anti-N-Cadherin (dilution 1:1000, #14215), mouse anti-Cytokeratin (dilution 1:1000, #4545), mouse anti-Vimentin (dilution 1:1000, #49636), rabbit anti-β-actin (dilution 1:5000, ab179467, Abcam, Cambridge, UK), rabbit anti-*EDEM1* (dilution 1:1000, ab200645), HRP-conjugated goat anti-rabbit rabbit (dilution 1:8000, ab6721), and goat anti-mouse rabbit (dilution 1:8000, ab6789) secondary antibody.

### *In vitro* scratch assay

Cells were seeded into 6-well plates (1 × 10^6^ cells/well). When the cell confluence reached 90%, a pipette tip was used to scratch a gap on the cell monolayer and cultures were observed at 0, 24, 48, and 72 h. The rate of closure was assessed from three independent experiments.

### Transwell migration and invasion assays

For cell migration and invasion (the upper chamber was coated with Matrigel) assays, a 24-well Transwell plate system (Cell BioLabs, Inc., San Diego, CA, USA) was used to measure the migration and invasive capabilities of both CRC and FHC cells. Matrigel was obtained from Gibco. The experimental protocol has been described previously in detail [27]. After seeding, waited 12 hours before subsequent fixation, staining, and photographing.

### Animal experiments

For the tumor xenograft model, LoVo cells overexpressing *LINC00485* (1 × 10^6^ cells in 50 μL of PBS) or control cells (equal loading volumes) were implanted subcutaneously into the flank regions of 8-week-old female nude BALB/c mice (a total of 12 mice; 6 mice per group). In a different set of experiments, LoVo cells (1 × 10^6^ cells in 50 μL) were subcutaneously injected into the dorsal surface of nude mice (a total of 12 mice; 6 mice per group), in which six randomly selected animals were intratumorally injected with *miR-581* antagomir (13 μg in a 100 μL volume per mouse) plus an equal volume of Lipofectamine 2000 once weekly. The mock group (n=6) received an equal volume of phosphate buffered saline (PBS) solution. Tumor diameters were measured every 7 days. The animals were sacrificed on day 42, and tumors were meticulously excised. The tissue volume was calculated using the formula: 0.5 × width^2^ × length.

To examine the ability of tumor cells to metastasize to the liver, LoVo cells (1 × 10^6^ cells in 50 μL) were injected into the spleen of mice (a total of 12 mice; 6 mice per group) under anesthesia. *MiR-581* antagomir (13 μg in volume of 100 μL per mouse) plus an equal volume of Lipofectamine 2000 was administered via tail vein once weekly. Another six animals received an equal volume of PBS plus Lipofectamine 2000. Additionally, we inoculated LoVo cells or LoVo cells overexpressing LINC00485 (1 × 10^6^ cells in 50 μL) into the spleen of nude mice (a total of 12 mice; 6 mice per group) as mentioned above. All animals were sacrificed on day 28, liver tissues were carefully removed and fixed with 10% formalin solution. Paraffin sections (5 μm) were prepared for hematoxylin-eosin (H&E) staining and all metastatic nodules were counted in the section. Mice used in this study were purchased from Model Animal Research Center of Nanjing University. Animal experiments were approved by the Institutional Animal Care and Use Committee of Nanjing First Hospital.

### Statistical analysis

Differences between two groups were assessed by applying student’s t-test. Multiple comparison was analyzed using the one-way analysis of variance (ANOVA) with Fisher's least significant difference (LSD) test. Survival analysis was analyzed using Kaplan-Meier survival curves with a log-rank test. Data are expressed as mean ± standard deviation (SD). P < 0.05 indicated that the difference was statistically significant. All *in vitro* experiments were performed in three independent experiments.

## Supplementary Material

Supplementary Figures
