# Supplementary material for: Long intergenic non-coding RNA LINC00485 exerts tumor-suppressive activity by regulating miR-581/EDEM1 axis in colorectal cancer
Source: Aging (Albany NY). 2021 Jan 10;13(3):3866–85. doi: 10.18632/aging.202354 (PMC7906134; doi:10.18632/aging.202354)
Supplement: Supplementary Figures [file aging-13-202354-s001.pdf]

SUPPLEMENTARY FIGURES

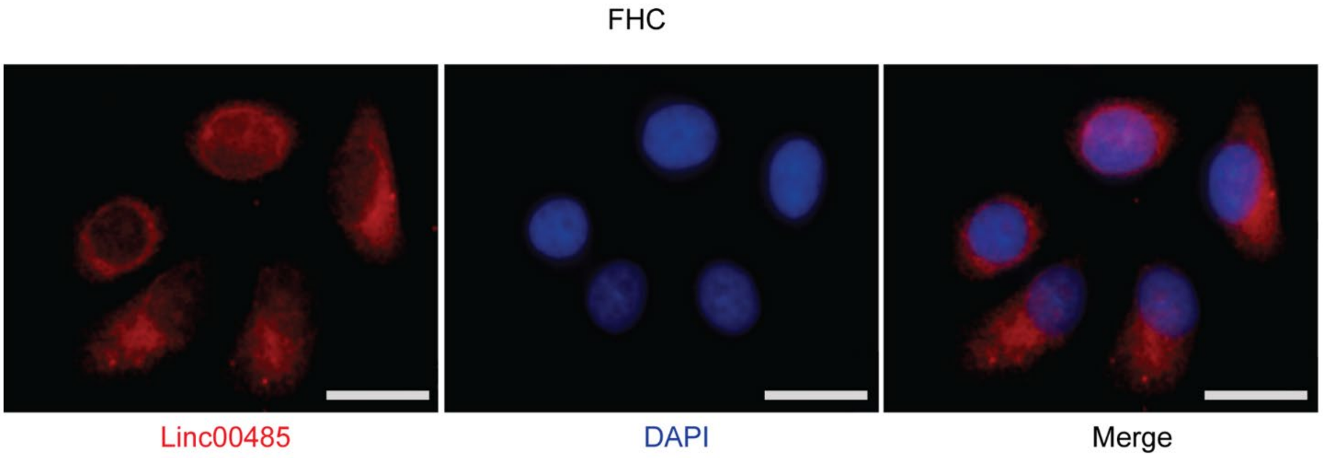

Supplementary Figure 1. The cellular localization of Linc00485 in FHC cell. FISH assay indicates that Linc00485 is mainly localized in the cytoplasm of FHC cells. Scale bar, 2 µm.

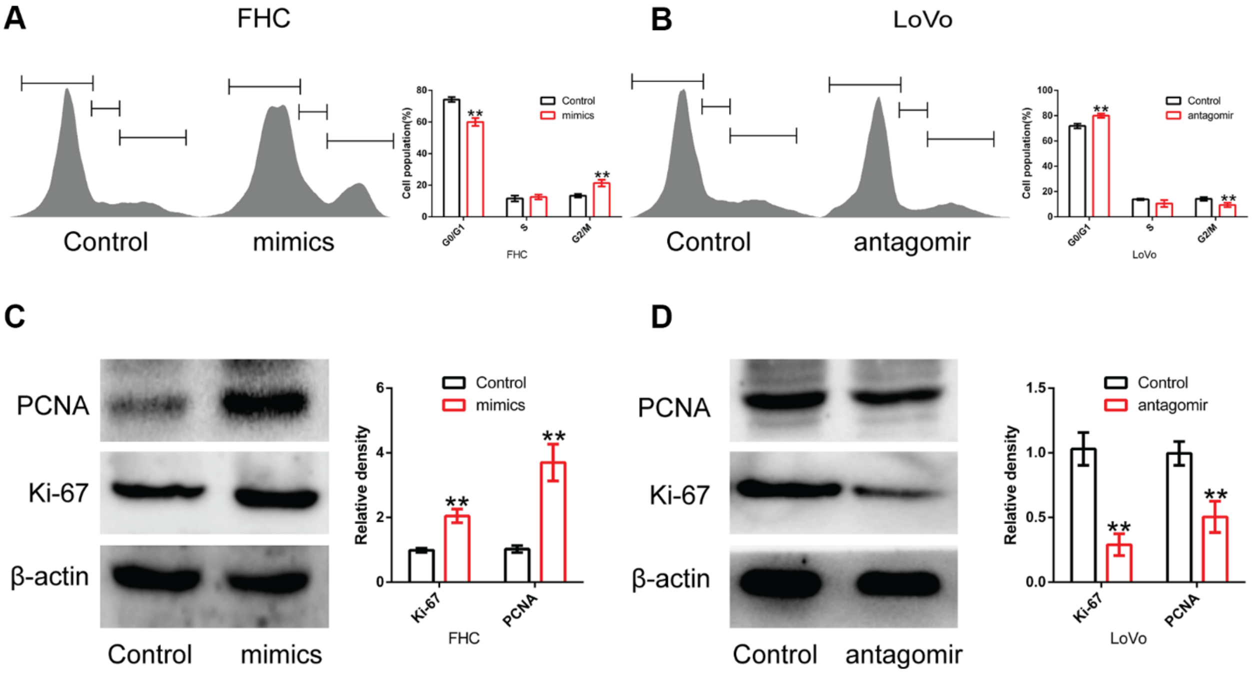

Supplementary Figure 2. The effects of miR-581 mimics/antagomir transfection on FHC or LoVo cells. (**A**) After transfection with miR-581 mimics, the cell cycle was arrested in G0/G1 phase in FHC cells. (**B**) After transfection with miR-581 antagomir, the cell cycle was arrested in G0/G1 phase in LoVo cells. (**C**) The overexpression of miR-581 up-regulated the protein expression levels of Ki-67 and PCNA in FHC cells. (**D**) miR-581 knockdown down-regulated the protein expression levels of Ki-67 and PCNA in LoVo cells. Data were analyzed using Student's test. Bars were represented as S.D. ***P*<0.01. PCNA, proliferating cell nuclear antigen.

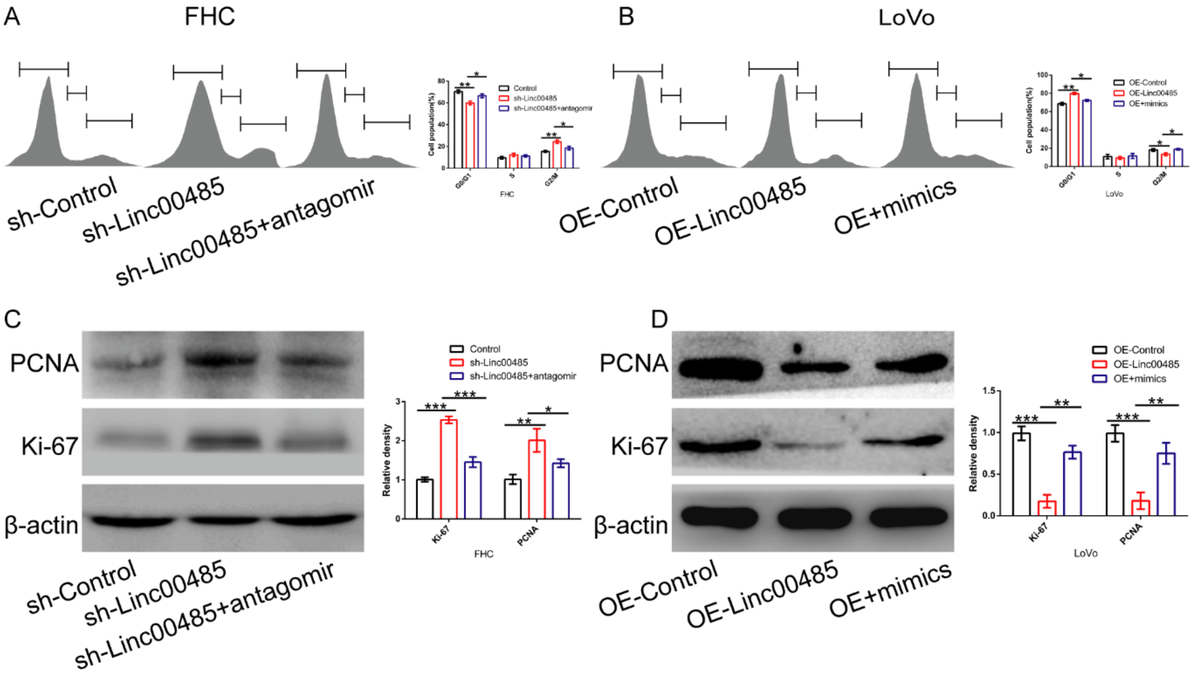

Supplementary Figure 3. Linc01088/miR-581 axis regulates cell cycle and the expression of proliferative indexes PCNA and Ki-67. (**A**, **B**) Cell cycle of (**A**) LINC00485 silenced FHC cells with or without miR-581 antagomir treatment for 24 h and (**B**) LINC00485-overexpressed LoVo cells with or without miR-581 mimics treatment for 24 h was measured by the Flow Cytometry. (**C**, **D**) The protein expression levels of PNCA and Ki-67 in (**C**) LINC00485 silenced FHC cells with or without miR-581 antagomir treatment for 24 h and (**D**) LINC00485-overexpressed LoVo cells with or without miR-581 mimics treatment for 24 h were detected by Western blotting assays. Data was analyzed using the one-way ANOVA with LSD test. Bars were represented as S.D. *P<0.05; **P<0.01; ***P<0.001. PCNA, proliferating cell nuclear antigen; sh, short hairpin RNA targeting LINC00485; OE, overexpression.

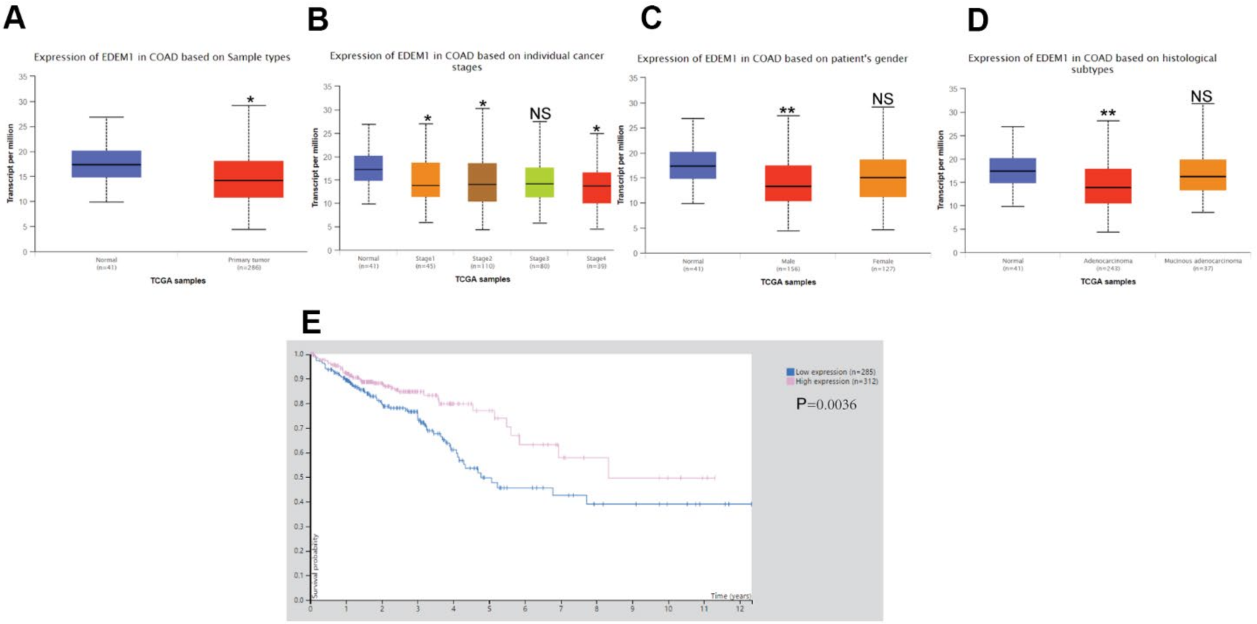

Supplementary Figure 4. The relationship of EDEM1 expression with clinical parameters of COAD patients in TCGA and HAP databases. Expression of EDEM1 in patients with COAD based on (**A**) sample types, (**B**) individual cancer stages, (**C**) patient's gender and (**D**) histological subtypes. (**E**) Low expression of EDEM1 predicted a poor prognosis of CRC patients. COAD, colon adenocarcinoma. *P<0.05; **P<0.01; NS, not significant.
